# PreDREM: a database of predicted DNA regulatory motifs from 349 human cell and tissue samples

**DOI:** 10.1093/database/bav007

**Published:** 2015-02-27

**Authors:** Yiyu Zheng, Xiaoman Li, Haiyan Hu

**Affiliations:** ^1^Department of Electrical Engineering and Computer Science and ^2^Burnett School of Biomedical Science, College of Medicine, University of Central Florida, Orlando, FL 32816, USA

## Abstract

PreDREM is a database of DNA regulatory motifs and motifs modules predicted from DNase I hypersensitive sites in 349 human cell and tissue samples. It contains 845–1325 predicted motifs in each sample, which result in a total of 2684 non-redundant motifs. In comparison with seven large collections of known motifs, more than 84% of the 2684 predicted motifs are similar to the known motifs, and 54–76% of the known motifs are similar to the predicted motifs. PreDREM also stores 43 663–20 13 288 motif modules in each sample, which provide the cofactor motifs of each predicted motif. Compared with motifs of known interacting transcription factor (TF) pairs in eight resources, on average, 84% of motif pairs corresponding to known interacting TF pairs are included in the predicted motif modules. Through its web interface, PreDREM allows users to browse motif information by tissues, datasets, individual non-redundant motifs, etc. Users can also search motifs, motif modules, instances of motifs and motif modules in given genomic regions, tissue or cell types a motif occurs, etc. PreDREM thus provides a useful resource for the understanding of cell- and tissue-specific gene regulation in the human genome.

**Database URL:** http://server.cs.ucf.edu/predrem/.

## Introduction

Identifying motifs of regulatory proteins and their cofactors in diverse cell or tissue types is critical for the global understanding of gene transcriptional regulation. A major type of regulatory proteins is sequence-specific DNA binding transcription factors (TFs), which modulate expression of their target genes by binding to short DNA segments called transcription factor binding sites (TFBSs) (1). TFBSs of a TF are in general similar. The common pattern of the TFBSs bound by a TF is called a motif, often represented as a consensus sequence or a position weight matrix (PWM) (2). In higher eukaryotes, multiple TFs often cobind short genomic regions of several hundred base pairs (bp) long and control the temporal and spatial expression patterns of target genes (3–8). A short genomic region with TFBSs of multiple TFs is called a cis-regulatory module (CRM) (3). Correspondingly, we define a motif module as a group of motifs with their TFBSs co-occurring in a significant number of short genomic regions (9, 10). Because of the critical roles of CRMs and TFs in gene transcriptional regulation, it is important to discover motifs of TFs and their cofactors.

Despite the existence of several public repositories of known DNA regulatory motifs (11–19), these repositories may miss motifs of a large number of active TFs in cell or tissue types under consideration. For instance, FactorBook and the collection by Wang *et al*. and Kheradpour and Kellis (12, 16) arguably have the most complete list of motifs in specific cell types, as they considered all TF-based chromatin immunoprecipitation followed by massive parallel sequencing (ChIP-seq) experiments in the Encyclopedia of DNA Elements (ENCODE) project (20). However, to date, there are only 86 and 124 TFs with available ChIP-seq datasets in GM12878 and K562, respectively, two of the most intensively studied cell types by the ENCODE project, in contrast with the existence of possibly several hundred active TFs in these cell types (21).

The DNase I hypersensitive sites (DHSs) sequencing (DNase-seq) provides an unprecedented opportunity to discover motifs of potentially all active sequence-specific DNA binding TFs under experimental conditions (22). DNase-seq identifies DHSs on the genome scale. DHSs are active regulatory regions that contain TFBSs of potentially all active TFs under an experimental condition (23). For instance, according to a recent study, 98% of TFBSs mapped by TF-based ChIP-seq experiments in ENCODE are located in DHSs (24). In contrast, a TF-based ChIP-seq experiment can identify only regulatory regions bound by one TF and some of its cofactors under a given condition. Therefore, DHS datasets are more viable for comprehensive motif discovery than individual TF-based ChIP-seq datasets under a given condition.

In Zheng *et al.* (25), we applied a recently developed tool, SIOMICS (10, 26), to predict DNA regulatory motifs and motif modules in DHSs from 349 human samples. In each DHS dataset, we predicted 845–1325 motifs and 43 663–20 13 288 motif modules. We clustered similar motifs from different datasets into 2684 non-redundant motifs. We validated these predicted motifs and motif modules by comparing them with known motifs, motifs of known interacting TFs, predicted motifs in ChIP-seq datasets in the same samples by other methods, etc. We found that more than 84% of predicted motifs are similar to known motifs, and 54–76% of known motifs in seven motif collections are similar to our predicted 2684 motifs. Moreover, more than 76% of predicted top motifs by a popular method Dreme (27) from ENCODE ChIP-seq datasets in GM12878 and K562 are included in our predicted motifs from DHSs in the two cell lines. In addition, on average, 84% of motif pairs corresponding to known interacting TF pairs from eight resources are included in our predicted motif modules. All these comparisons suggest the near-comprehensiveness of our predicted motifs of potentially active sequence-specific DNA binding TFs and their active cofactors in the 349 samples.

Here we present PreDREM, a database storing the aforementioned predicted motifs and motif modules (25). PreDREM will be beneficiary to several types of hypothesis generating. First, PreDREM can help the study of a specific TF, with the information about tissue types the motif of this TF occurs, cofactor motifs this motif having in different tissues, links to this motif in public databases, etc. Second, PreDREM can help the study of TF interactions. Users can find motifs of cofactors that interact with a TF in different tissues, links to such interactions in public databases, genomic regions such interactions occur, etc. Third, PreDREM can help the study of individual genes, with predicted TFBSs in different tissues, potential TFs behind these TFBSs, together with other information such as TFBS conservation and DHS signals around TFBSs in public databases, etc. Fourth, PreDREM will be useful for the understanding of gene transcriptional regulation across tissue and cell types, with the predicted motifs and motif modules across 349 tissue and cell types. PreDREM is thus not only a repository of motifs and motif modules but also a good resource to understand tissue- and cell-specific gene transcriptional regulation in the human genome. PreDREM is freely accessible at http://server.cs.ucf.edu/predrem/.

## Materials and methods

### Workflow to discover motifs in PreDREM

**Figure 1.**
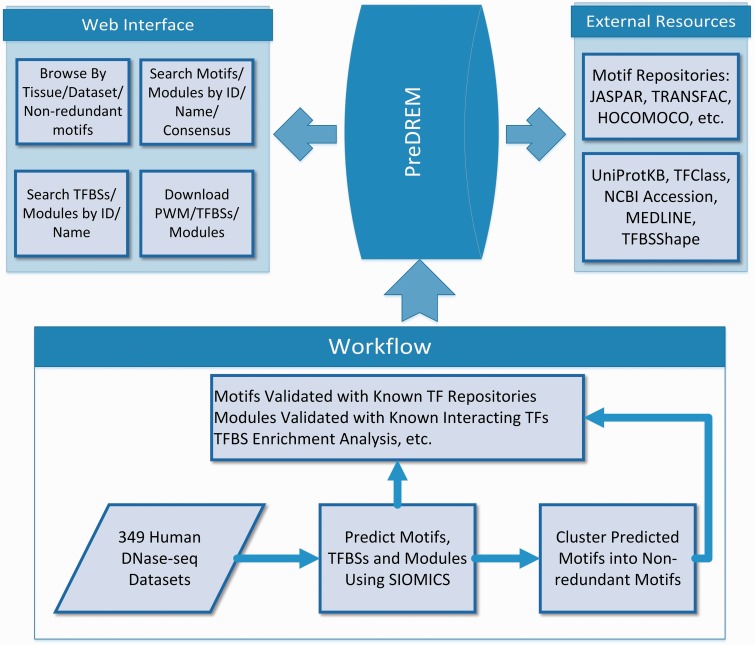
Overview of the PreDREM database.

The workflow to identify motifs and motif modules in 49 DHS datasets has been described previously (25). In brief (Figure 1), DHS regions from the 349 samples are downloaded from Ref. (23). DHSs longer than 5000 bp are removed, as they on average only account for <3.2% of DHS regions in a sample while significantly increase the time cost for motif discovery. Moreover, it is not statistically meaningful to consider the co-occurrence of multiple motifs in such long regions. We also extend DHSs shorter than 800 bp evenly from their two ends to 800 bp, because CRMs are on average 635 bp long (4) while the original DHSs are on average from 336–756 bp long in the 349 samples, and we do not intent to change the original DHSs much. SIOMICS (26) is then applied to repeat-masked sequences from these pre-processed DHSs in each dataset to predict motifs and motif modules. The motifs predicted in each dataset are called adjusted motifs, which are grouped into 2684 non-redundant motifs based on motif similarities. The predicted motifs and motif modules are validated using known motifs and motif pairs corresponding to the known interacting TF pairs.

### Validation of the predicted motifs and motif modules

We validated our prediction in PreDREM from several aspects. First, we studied motifs predicted by SIOMICS in random DHS datasets. We generated two random DHS datasets by permuting the DNA sequences from original DHSs in GM12878 and K562. Here, a permuted sequence was a random reorganization of nucleotides in the corresponding original sequence. Therefore, the two random DHS datasets had the same lengths and the same number of sequences as what we analyzed in GM12878 and K562 (25). We then applied SIOMICS to the two random datasets. We found only 11 and 20 motifs in the two random datasets, compared with 961 and 953 predicted motifs in the corresponding real DHS datasets. Second, we compared the predicted motifs and motif modules with known motifs and motif pairs of known interacting TF pairs. We found that more than 84% of the 2684 predicted motifs were similar to known motifs. Moreover, 54–75% of known motifs were similar to the 2684 predicted motifs compared with <6% of known motifs similar to 2684 randomly generated motifs (25). Third, we further compared our predicted motifs with the predicted top 5 motifs by Dreme in TF-based ChIP-seq datasets in GM12878 and K562. Dreme is a recently developed popular method for motif discovery in ChIP-seq datasets. We found that 76 and 81% of the predicted top motifs by Dreme in ChIP-seq datasets were included in our predictions in the two corresponding DHS datasets (25). Finally, we compared the predicted motif modules with motifs of known interacting TFs from eight resources, and found on average, 84% of motif pairs corresponding to known interacting TFs were included in our predicted motif modules. The details of these comparisons together with others are in (25) and the summary is on the statistics page in PreDREM.

## The PreDREM database

### Database overview

The PreDREM Database is organized to provide easy and user-friendly access to the predict motifs and motif modules. It is constructed on a Linux server running the MySQL database and the Apache HTTP Server. Apache Tomcat is used to serve as the container for servlet and JavaServer Pages (JSP). The front-end pages are implemented by JSP to provide a dynamic and functional web interface. A SQLite format database file is made available for download, which mainly contains the position frequency matrix of each motif and the datasets each motif occurs. It can be used directly for query or manipulated in R using TFBSTools (28).

The PreDREM database includes 845–1325 adjusted motifs in each of the 349 DHS datasets. It also contains 2684 non-redundant motifs resulted from clustering similar adjusted motifs across datasets. Based on the validation using known motifs, the reference information of each motif and the corresponding TFs are reported. For each motif, the reference information includes the corresponding UniProtKB entry (29), the classification of the corresponding TF in TFClass (30), the accession entry of the corresponding TF at NCBI, the link to the relevant publication reporting the TFBSs used in the motif model of the corresponding JASPAR motif and a link to the DNA shape features for TFBSs of the corresponding JASPAR motif (31). Moreover, for each adjusted motif, the significance of the enrichment of its TFBSs in certain types of genomics regions are calculated and reported in PreDREM (25). In addition, the motif modules and the corresponding known TF-TF interactions containing this adjusted motif are also reported in PreDREM.

Users can browse motifs and motif modules discovered in each dataset and in any given genomic region. Users can also search sequence-specific DNA binding TFs to see which tissue or cell types the TFs may be active and with which cofactors they may work in these tissues or cell types. Users are able to search TFBSs of motifs and motif modules in specified genomic regions in diverse cell or tissue types. Moreover, users can download adjusted motifs, non-redundant motifs, known motifs similar to predicted motifs, TFBSs of each motif, the enrichment of TFBSs of a motif in certain types of genomic regions, motif modules, motif modules supported by known TF-TF interactions, etc. See the download page in PreDREM for the full list of items that can be downloaded.

### The Browse interface

The ‘Browse’ page of the PreDREM interface allows users for a quick navigation of tissues, datasets and non-redundant motifs included in the database. Users can view a list of datasets related to a cell or tissue type, or browse all motifs discovered in one dataset by selecting the dataset, or directly visit the information page of any non-redundant motif.

**Figure 2.**
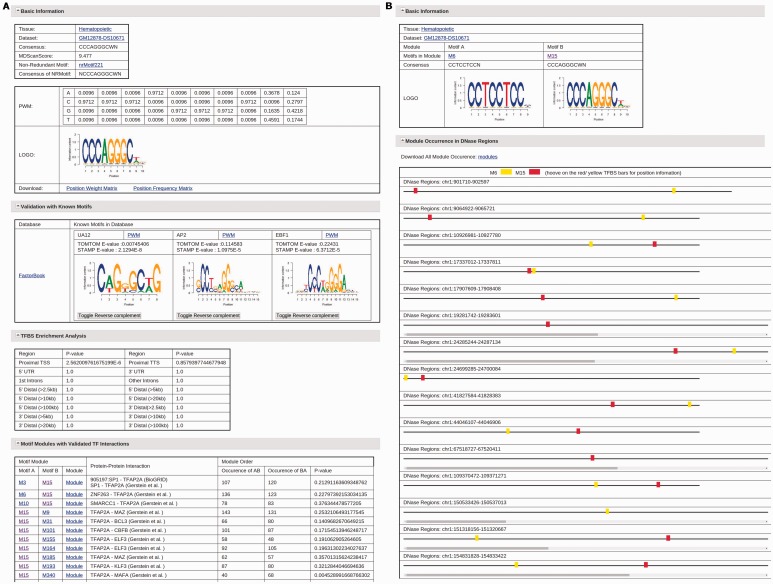
(**A**) The information page for the motif M15 in the dataset GM12878-DS10671. (**B**) The information page for the motif module composed of motifs M6 and M15 in the dataset GM12878-DS10671.

For each motif, the detailed information about this motif can be obtained by clicking the name of this motif (Figure 2A). This detailed information page for an adjusted motif in each dataset contains the following content: i) The basic information about the motif including cell and tissue type the motif occurs, datasets the motif occurs, the motif consensus, the motif PWM, the motif logo and the corresponding non-redundant motif; ii) The validation information with known motifs in seven motif databases. Similar known motifs from each repository with PWM, sequence logo, TOMTOM and STAMP comparison E-value are shown in the validation table. The external references to this validated motif in UniProtKB, TFClass, the accession number in NCBI, the references in MedLine and TFBSShape are listed when available; iii) The types of genomic regions where TFBSs of this motif are enriched and the corresponding enrichment *P*-values; iv) The list of motif modules that contain this motif and are validated by known TF interaction in eight resources. The preferred order of TFBSs of two motifs in a validated motif module is also listed, including how many times motif A are found to occur before motif B and vice versa. A significance *P*-value is given to each module to facilitate users to choose motif modules for further studies. Each module also has a corresponding link to its detailed information, which contains the information of the motifs in the module, and a visual chart of all occurrences of this motif module in DHSs (Figure 2B). Users can also download the list of all occurrences of the motif module with their TFBSs under given conditions., The complete list of motif modules discovered for each motif in each dataset, which may not be validated by known interacting TF pairs, can be found in the download page. The detailed information page for non-redundant motifs is similar, except that a table listing all adjusted motifs that result in this non-redundant motif replaces the TFBS and motif module analysis part.

### The Search Motifs/Modules page

The ‘Search Motifs/Modules’ page allows users to search motifs similar to known motifs in JASPAR, TRANSFAC and HOCOMOCO by searching the corresponding identifiers or TF names in these repositories. For instance, if users want to search all motifs similar to NFKB1, users can select ‘Search Motifs’ or ‘Search Non-Redundant Motifs’, and enter the corresponding identifiers: MA0105.3 in JASPAR, M00054 in TRANSFAC, or the TF name ‘NFKB1’. Users can also search motifs based on motif consensus. In the case of NFKB1, users can search ‘AA*TTTCC’. All motifs whose consensus contains both ‘AA’ and ‘TTTCC’, and ‘AA’ occurs before ‘TTTCC’ will be returned. It is worth mentioning that both fuzzy TF name search and wild card in consensus search are enabled. That is, users do not need to input the full name of a TF. For instance, to search with the keyword ‘Pax’ will return all motifs of TFs of the PAX family. When users try to search motifs based on consensus, the wild character (*) can be at any place of the keyword. The search result will contain all possible motifs that match the keyword in all datasets. ‘Match’ is defined as meeting any of the following three criteria: i) ID of any of the most similar TFs in three external motif databases (JASPAR, TRANSFAC and HOCOMOCO) is identical to the input keyword; ii) name of any of the most similar TFs in the three motif databases contains the input keyword; iii) consensus of the motif matches the input keyword.

Users can also search validated motif modules in the same page by selecting the ‘search modules’ option. If only one ID or name is input as the search keyword, the output will be all validated motif modules containing at least one motif matching the keyword. If two or more IDs/names are used in the search keyword, the search result will be motif modules that are composed of motifs matching two of the input IDs/names. In the case of searching motif modules containing NFKB1 and SP1, users simply need to input ‘NFKB1 SP1’ or the corresponding identifiers in the three repositories (JASPAR, TRANSFAC and HOCOMOCO). The module search result will only contain validated motif modules by known interacting TFs currently. The full list of motif modules, which are the supersets of the validated modules, can be found in the download page.

### The Search TFBSs/Modules page

The ‘Search TFBSs/Modules’ page allows users to query TFBSs of the selected motifs in specified genomic regions in one of the 349 DHS datasets. After users select a dataset, all motifs discovered in this dataset will be listed in the ‘Motif Selection’ panel. Users can then choose multiple motifs of interest. To obtain more information about each motif, users can hover the mouse pointer over any motif, and the names of the corresponding motifs in HOCOMOCO, JASPAR and TRANSFAC will be shown. Users can also filter motifs by inputting identifiers or TF names following similar keyword criteria as those in ‘Search Motifs/Modules’. Only motifs related to the searching keyword will be left in the ‘Motif Selection’ panel. Upon the submission of a query, users will receive the list of TFBSs related to the selected motifs in restricted genomic regions as a file in BED format.

**Figure 3.**
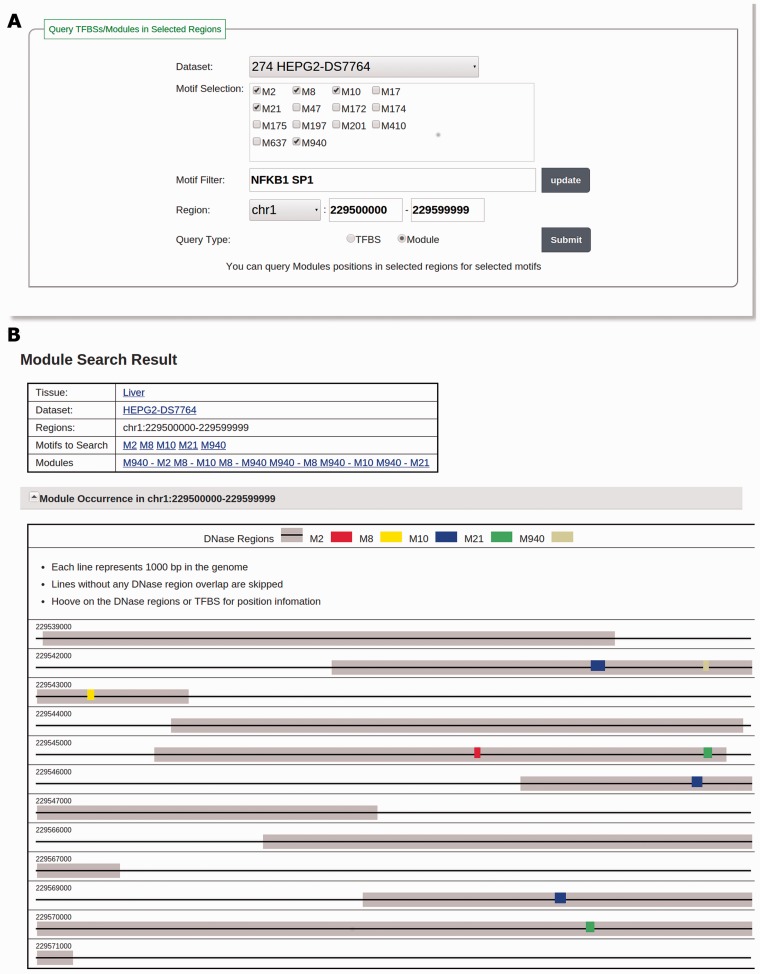
(**A**) The input page for searching modules with the keyword ‘NFKB1 SP1’ in the dataset HEPG2-DS7764 and in the region of ‘chr1:229500000–229599999’. The motif selection panel shows adjusted motifs discovered in HEPG2-DS7764 filtered by keyword ‘NFKB1 SP1’. One motif related to NFKB1 (M950) and four motifs related to SP1 (M2, M8, M10 and M21) are selected. (**B**) The result page of the searching in A. For the selected motifs, six motif modules composed of them are found and listed in the ‘Modules’ section. TFBSs of these motifs in DHSs are visualized in the ‘Module Occurrence’ section.

Users can also search TFBSs of motifs in validated motif modules by changing the query type to ‘Module’ and follow the same selection method as that to search TFBSs. Figure 3A illustrates how to search motif module instances for NFKB1 and SP1 in the dataset ‘HEPG2-DS7764’ in the region ‘chr1:229500000–229599999’. The database will return a list of validated motif modules that are related to the selected motifs and a visualization of all TFBSs of the motifs in these validated motif modules in the specified genomic regions (Figure 3B).

## Future development

Several new features will be added to PreDREM in the near future. An enforced search of TFBSs and modules will be added, allowing searching in multiple datasets and multiple genomic regions. Also, the algorithm to cluster motifs is under constant refinement to further improve the quality of non-redundant motifs. In addition, we will enable to search all predicted motif modules instead of the subset of the predicted motif modules that are validated by TF interactions. Finally, computationally predicted target genes of TFBSs and motif modules will be added to replace the current information of closest upstream and downstream genes listed in the TFBS information page for each motif.

## Summary

The PreDREM database allows users to browse and search predicted motifs and motif modules of potentially active sequence-specific DNA binding TFs in 349 cells and tissues. The predicted motifs are ranked and validated with known motifs in other databases, and the predicted motif modules are validated with known TF interactions to facilitate further research. PreDREM provides a unique resource for the understanding of gene transcriptional regulation in different cell and tissue types.

## Funding

This work is supported by the National Science Foundation (grants 1149955, 1356524 and 1218275). Funding for open access charge: The National Science Foundation (grant 1149955 from the Division of Biological Infrastructure).

*Conflict of interest.* None declared.
